# We and herbivores eat endophytes

**DOI:** 10.1111/1751-7915.13688

**Published:** 2020-12-15

**Authors:** Esperanza Martínez‐Romero, José Luis Aguirre‐Noyola, Rafael Bustamante‐Brito, Pilar González‐Román, Diana Hernández‐Oaxaca, Víctor Higareda‐Alvear, Leslie M. Montes‐Carreto, Julio César Martínez‐Romero, Mónica Rosenblueth, Luis Eduardo Servín‐Garcidueñas

**Affiliations:** ^1^ Programa de Ecología Genómica Centro de Ciencias Genómicas UNAM Cuernavaca Morelos Mexico; ^2^ Laboratorio de Microbiómica Escuela Nacional de Estudios Superiores Unidad Morelia UNAM Morelia Michoacán México

## Abstract

Health depends on the diet and a vegetal diet promotes health by providing fibres, vitamins and diverse metabolites. Remarkably, plants may also provide microbes. Fungi and bacteria that reside inside plant tissues (endophytes) seem better protected to survive digestion; thus, we investigated the reported evidence on the endophytic origin of some members of the gut microbiota in animals such as panda, koala, rabbits and tortoises and several herbivore insects. Data examined here showed that some members of the herbivore gut microbiota are common plant microbes, which derived to become stable microbiota in some cases. Endophytes may contribute to plant fibre or antimetabolite degradation and synthesis of metabolites with the plethora of enzymatic activities that they display; some may have practical applications, for example, *Lactobacillus plantarum* found in the intestinal tract, plants and in fermented food is used as a probiotic that may defend animals against bacterial and viral infections as other endophytic‐enteric bacteria do. *Clostridium* that is an endophyte and a gut bacterium has remarkable capabilities to degrade cellulose by having cellulosomes that may be considered the most efficient nanomachines. Cellulose degradation is a challenge in animal digestion and for biofuel production. Other endophytic‐enteric bacteria may have cellulases, pectinases, xylanases, tannases, proteases, nitrogenases and other enzymatic capabilities that may be attractive for biotechnological developments, indeed many endophytes are used to promote plant growth. Here, a cycle of endophytic‐enteric‐soil‐endophytic microbes is proposed which has relevance for health and comprises the fate of animal faeces as natural microbial inoculants for plants that constitute bacterial sources for animal guts.

## Graphical abstract

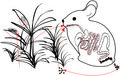

## Effects of vegetal diet and endophytes

### Vegetal diet

Vegetables provide fibres, vitamins and metabolites that promote health (Cardona *et al*, 2013; Klinder *et al*, 2016; Makki *et al*, 2018), but their role as microbe providers is less known, unless these microbes are pathogens. When animals consume raw plants, they eat their associated bacteria. Eating an apple may provide hundred millions of bacteria (Wassermann *et al*, 2019), as does eating bananas, lettuce (Berg *et al*, 2014a) or other raw vegetables and non‐pasteurized juices. Even if vegetables are washed, peeled or disinfected, they still provide microbes because the endophytic bacteria or fungi reside in the plant interior protected from disinfectants. Herbivore guts had the largest diversity of bacteria, containing 14 phyla, while only six phyla were found in carnivores (Ley *et al*, 2008). Different species of *Bifidobacterium* and *Lactobacillus* were found in herbivores compared with carnivore or omnivore animals (Endo *et al*, 2010). The gut human microbiota has been extensively studied (reviewed in Thursby and Jurge, 2017; Rothschild *et al*, 2018) and depends on the diet (Muegge *et al*, 2011; David *et al*, 2014). Transient microbiota (called foreign microorganisms by David *et al*, 2014) may derive from food. Notably, the gut microbiome in humans is determined by the number of vegetables consumed (McDonald *et al*, 2018). Ingested bacteria may be metabolically active in human guts as revealed by gene transcripts from food‐bacteria in guts (David *et al*, 2014). Furthermore, plant‐borne pathogens provide an unfortunate example of human ingestion of plant bacteria (Berg *et al*, 2014a, 2014b; Rosenblueth and Martinez‐Romero, 2006). Certainly, plant bacteria have been in our diet for a long time (Berg *et al*, 2014a, 2014b) and for the whole evolutionary history of herbivores. The evolutionary history of insects is tightly dependent on plants as food (McKenna and Farell, 2006) and large radiations in insects followed plant diversification (Futuyma and Agrawal, 2009).

Besides containing bacteria, plants may modify gut bacterial composition and diversity due to their content of fibres, flavonoids, carotenoids, alkaloids, bioactive metabolites, antimetabolites or toxins (Cardona *et al*, 2013; Klinder *et al*, 2016; Makki *et al*, 2018; Baxter et al., 2019). Japanese that eat seaweeds have a peculiar microbiota (Hehemann *et al*, 2010).

### Endophytes

All plants in nature and crops have associated microbes (Friesen *et al*, 2011) in apparently all organs and tissues. Microbes that colonize inner plant tissues are designated endophytes (Rosenblueth and Martinez‐Romero, 2006; Harrison and Griffin, 2020; Berg *et al*, 2014a, 2014b), as the Greek‐prefix ‘endo’ means inside or within and ‘phyton’ means plant. Endophytes are a selected group of plant‐associated microbes (Rosenblueth *et al*, 2004; Rosenblueth and Martinez‐Romero, 2006; Hardoim *et al*, 2015; Busby *et al*, 2016) in the sense that only particular microbial genotypes are capable of internally colonizing specific plants. Endophytes promote plant growth by different strategies, such as suppressing or out‐competing pathogens, fixing nitrogen, producing hormones that stimulate plant growth, protecting from stress or enhancing the availability of minerals (Rosenblueth and Martínez‐Romero, 2006).

## Endophytes in animal guts

Gut microbiota may contain hundreds to thousands of bacterial species (McDonald *et al*, 2018; Rothschild *et al*, 2018) that may be acquired from or selected by the diet. Bacterial cultures from herbivore faeces showed bacteria that are evidently derived from plants, for example methylobacteria isolated in cultures from rhino (*Rhinoceros sondaicus*) and alpaca faeces (Jiang *et al*, 2013) and detected in cabbage white butterfly (Robinson *et al*, 2010b). Methylobacteria (commonly found in plants) use methanol, a sub‐product of plant cell wall biosynthesis and produce the plant hormones cytokinins (Lidstrom and Chistoserdova, 2002). Furthermore, cultured actinobacteria from herbivores showed that *Streptomyces, Rhodococcus* and *Microbacterium* were the dominant isolates from all six animal faeces tested, including an elephant (Jiang *et al*, 2013). These bacteria are common plant endophytes, for example, *Microbacterium* was isolated from legume nodules in arid regions (Zakhia *et al*, 2006) and from a halophyte in a salt‐marsh (Alves *et al*, 2014), *Streptomyces* was found as a seed endophyte in a *Phaseolus vulgaris* (common bean) cultivar with outstanding characteristics (López‐López et al 2010) and a *Rhodococcus* leaf endophyte enhanced resistance to pathogenic fungi in potato (Hong *et al*, 2016).

**Fig. 1:**
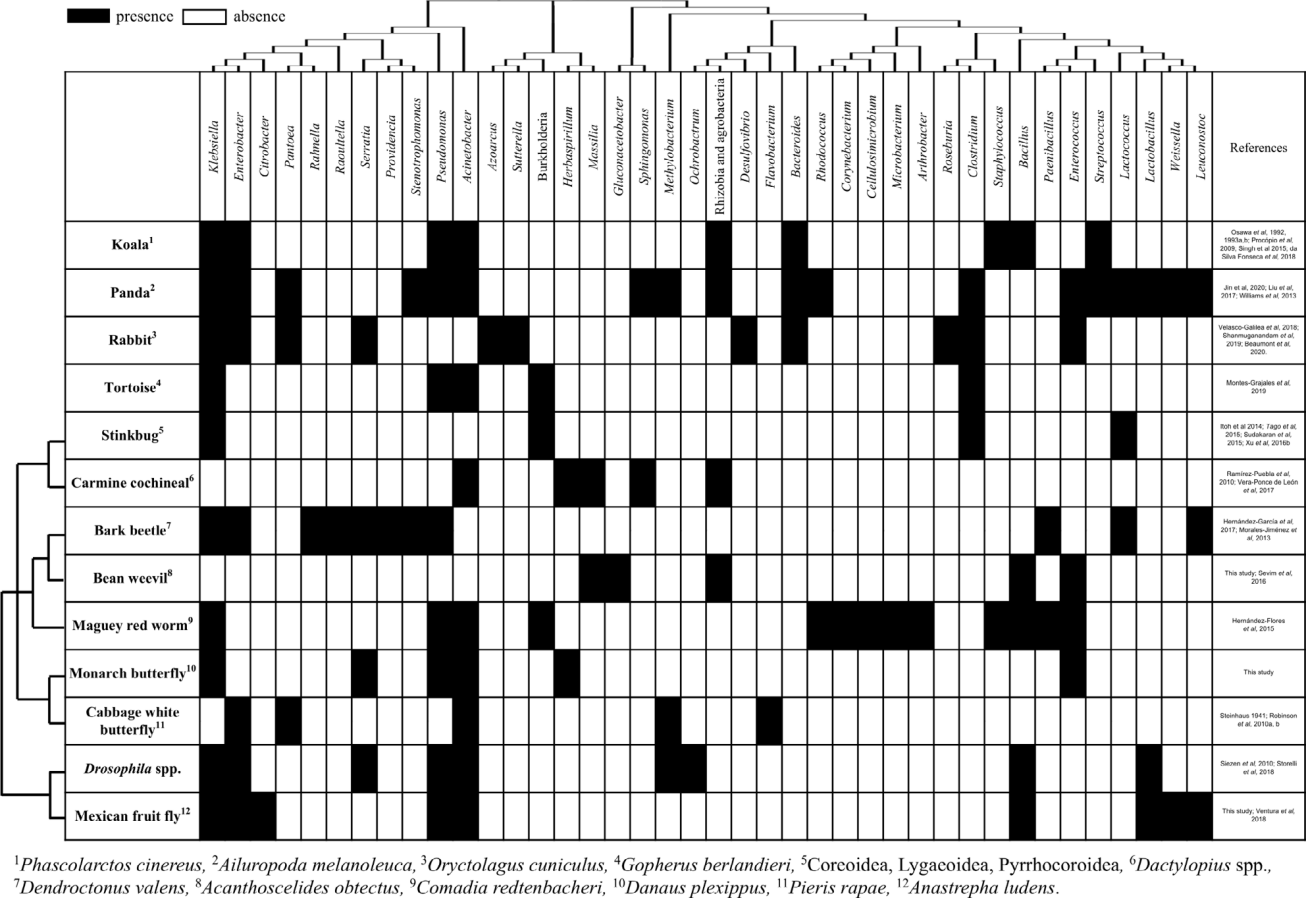
Examples of animal gut bacteria (from cultures or identified in gut metagenomes) that are common plant endophytes.

**Fig. 2:**
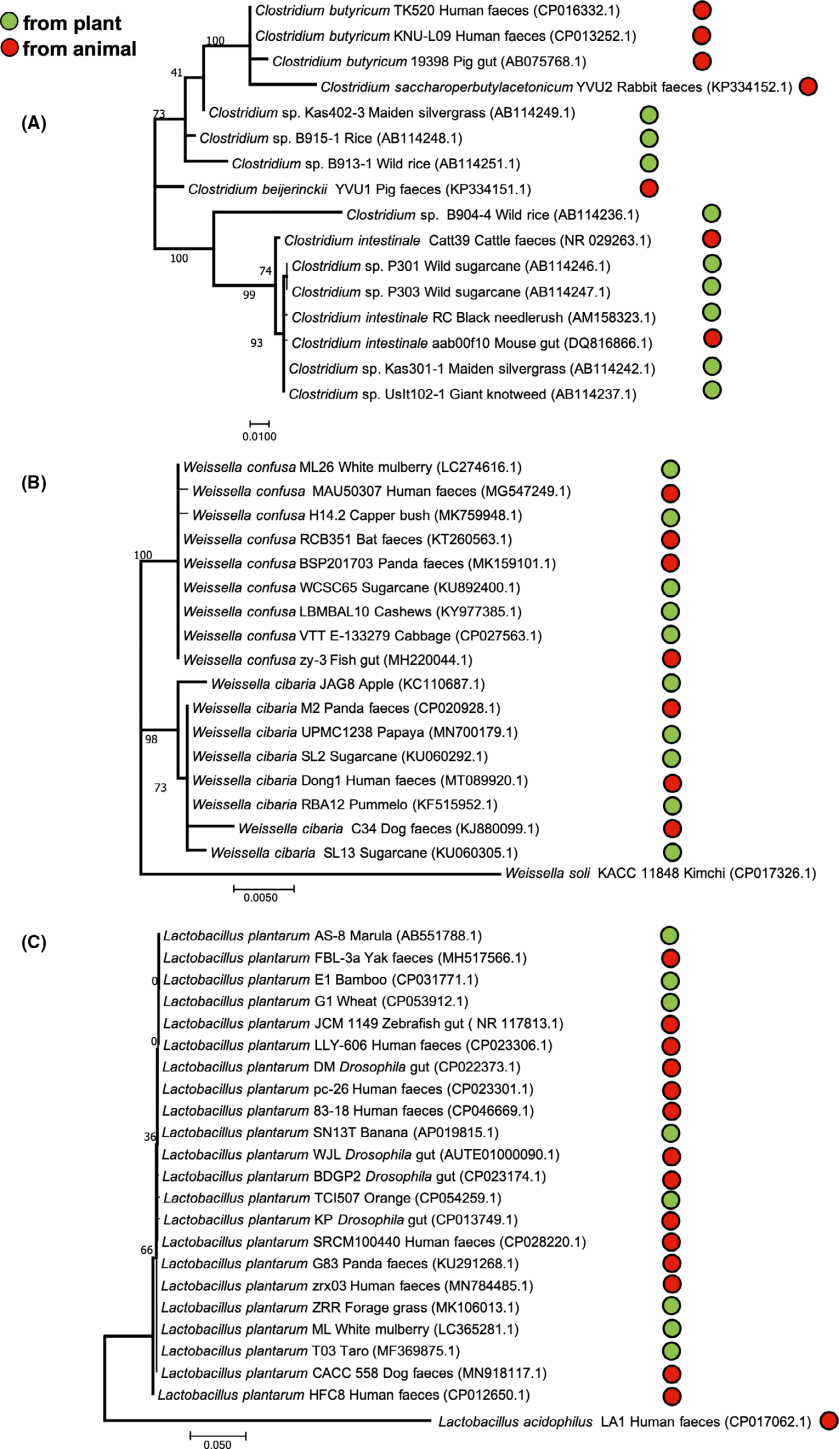
16S rRNA gene phylogenies of gut bacteria and endophytes of selected genera. A. *Clostridium . B. Weissella. C. Lactobacillus plantarum .* 16S rRNA sequences from NCBI database were aligned using Clustal W and phylogenetic trees were constructed in MEGA X software using the maximum likelihood method and general time reversible model with 1000 bootstraps replicates. A total of 1369 positions were included for the phylogenetic analysis.

Endophytes that feed on plants would have fibre degrading capabilities that would help animals degrade plant polysaccharides in guts. Plant fibres containing cellulose and hemicellulose are not easily digested, and sets of large numbers and diverse degrading enzymes are needed, including glycoside hydrolases and polysaccharide lyases, which are not produced or rarely produced by mammals (El Kaoutari *et al*, 2013). *Clostridium* is found as an endophyte and in many animal guts (Figs 1 and 2). Clostridial cellulolytic activity is remarkable because these bacteria may contain cellulosomes (Bayer and Lamed, 1986; Schwarz, 2001), which are considered the most efficient natural nanomachines (Nunes, 2018) that degrade both cellulose and hemicellulose. Cellulosomes are protuberances on the bacterial cell wall (Bayer and Lamed, 1986) containing complex enzymatic systems. *Enterobacter* that is found in herbivore guts or faeces and in plants (Fig. 1) has diverse polysaccharide degrading enzymes such as cellobiosidase, endoglucanase, polygalacturonase, xylanase, β‐glucuronidase, pectinases and cellulases (Prem Anand *et al*, 2010; Naveed *et al*, 2014; Xia *et al*, 2017). Cellulases are found in many Proteobacteria, Firmicutes and Actinobacteria (Berlemont and Martiny, 2013).

In contrast to fibre or wood‐chewing insects, sap‐sucking insects such as stinkbugs or cochineals may have bacteria with less fibre degrading capabilities. In sap‐sucking insects, protease activity was found in guts and it was suggested that ‘digestive proteolysis may be widespread in homoptera’ (Foissac *et al*, 2002). In the carmine cochineal, *Dactylopiibacterium* showed increased expression of protease and peptidase genes in a gut metatranscriptomic analysis suggesting a bacterial origin of proteases in guts (Bustamante‐Brito *et al*, 2019).

Some herbivores have a specialized diet, for example koalas eat eucalyptus, pandas eat bamboos, tortoises eat cactus, Monarch butterfly pupas eat *Asclepias* and maguey red worms eat *Agave* cactuses and their microbiota serves to digest some of the particular substances or antimetabolites in their host plants. Some of the antimetabolites may be degraded in guts by endophytes such as *Pseudomonas,* burkholderias and *Enterobacter* (Shanmuganandam *et al*, 2019). Tannins are the fourth more abundant plant molecules after cellulose, hemicellulose and lignin, with tannic acid as the most abundant reserve in plants (de Las Rivas *et al*, 2019). Tannases that degrade tannins found in eucalyptus, quercus and other tree leaves ingested by herbivores, are produced by many bacterial genera found in guts and plants, including *Enterobacter, Weissella* and *Lactobacillus* (de Las Rivas *et al*, 2019). Pinene, from pine trees that are ingested by different beetles, is degraded by some *Pseudomonas* using monooxygenases, lyases and aldehyde dehydrogenases (Linares *et al*, 2009).

The importance of endophytic fungi in plants and ecosystems is well appreciated (Harrison and Griffin, 2020). In turn, fungi may harbour bacterial communities that would also have an impact in plants (Bonfante *et al*, 2019). We suppose that from the outstanding diversity of fungi in plants (Harrison and Griffin, 2020) only a few fungi colonize animal guts. Some fungi are commonly found inside insects and plants (Pažoutová *et al*, 2013; Chen *et al*, 2018; Biederman and Vega, 2020) and may have cellulases and tannases (Martin, 1992; de Las Rivas *et al*, 2019). The most common contribution from fungi to their insect symbiont is the catalytic capacity to break down plant polysaccharides such as cellulose and pectin from their diet (Martin, 1992). Herrera *et al* (2011) found sequences 97% similar to root‐associated fungi in coprophilous fungal communities obtained from the dung of four species of mammalian herbivores. The effects of eating endophytic fungi were observed with sheep. Sheep were fed ryegrass with or without distinct fungal endophytes, and later sheep faeces were studied for their degradation rate. Faeces from animals that ate grass with fungal endophytes had the lowest faecal degradation rates (Cripps *et al*, 2013). This nice example showed that fungal endophytes from grasses that were consumed by sheep arrived into guts and exerted effects on faeces.

Insects among animals seem to have had unique associations with fungi for around 420 million years. These associations range from pathogenic with *Cordyceps* for example, to obligate mutualisms found in beetles, wasps and ants (Boucias *et al*, 2012a, 2012b; Gibson and Hunter, 2010). Remarkably, some of the insect‐associated fungal groups are well‐known endophytes within the Sordariomycetes and Agaromycetes (Naranjo‐Ortiz and Gabaldón, 2019).

Interestingly, some protists may inhibit cellulolytic activities in guts (Ray *et al*, 2012). Protists and archaea though they are associated with plants and guts, will not be revised here, neither the rumen or bee microbiota (Taxis *et al*, 2015; Engel *et al*, 2012; Zheng *et al*, 2019 Raymann *et al*, 2018; Motta *et al*, 2018. Powell *et al*, 2018; Raymann and Moran, 2018; Leonard *et al*, 2020). Previously, a deep analysis of anaerobic adaptations of bacteria in arthropod guts and the possible DNA contaminants in reagents were reported (Degli Esposti and Martinez‐Romero, 2017; Glassing *et al*, 2016). We show here examples of gut bacteria with a possible endophytic origin (Fig. 1), detected in pandas, koalas, rabbits, tortoises and several insects that are reviewed below.

### Koala

The koala (*Phascolarctos cinereus*) has a diet based almost exclusively on *Eucalyptus* leaves (Moore and Foley, 2000). Secondary metabolites contained in *Eucalyptus* plants act as toxins and antimicrobial agents that could affect the koala and its microbiota (Moore *et al*, 2004; Brice *et al*, 2019). The koala gut microbiota is highly conserved and specialized in the digestion and detoxification of dietary components (Brice *et al*, 2019). Blyton *et al* (2019) demonstrated that oral‐faecal inoculation between wild koalas with different feeding habits allows them to feed on *Eucalyptus* species that were previously inedible. Similarly, gut microbiota are acquired by juvenile koalas when they feed on special maternal faeces called 'pap' (Osawa *et al*, 1993a, 1993b). The strict vegetarian diet of koalas leads to a constant supply of endophytic microbes, which may become resident or transitory inhabitants of their guts. The koala rectum is mainly colonized by Bacteroidetes, Firmicutes and Proteobacteria (Barker *et al*, 2013; Alfano *et al*, 2015). *Staphylococcus*, *Bradyrhizobium* and *Acinetobacter* have been found in koala guts and as *Eucalyptus* endophytes (Procópio *et al*, 2009; da Silva Fonseca *et al*, 2018). Likewise, tannin‐degrading strains of *Streptococcus*, *Enterobacter* and other Enterobacteriaceae have been isolated from koala faeces (Osawa, 1992), 'pap' (Osawa *et al*, 1993a, 1993b) and *Eucalyptus* leaves (Miguel *et al*, 2016). Cellulose‐degrading *Pseudomonas* and spore‐forming bacilli are other examples of microbiota shared between koala faeces and *Eucalyptus* tissues (Singh *et al*, 2015; Miguel *et al*, 2016).

### Giant panda

The giant panda (*Ailuropoda melanoleuca*) consumes about 15 kg of leaves, stems and shoots of bamboo every day (Dierenfeld *et al*, 1982). Due to their carnivorous origin, pandas have a straight short gastrointestinal tract (Ley *et al*, 2008) whose microbiota is rich in Proteobacteria and Firmicutes (Fig. 1) (Tun *et al*, 2014; Xue *et al*, 2015; Guo *et al*, 2019). However, the gut microbiota is affected depending on the part and species of bamboo consumed. For instance, a high leaf consumption leads to an increase in *Bacteroides* and a decrease in *Lactobacillus,* but it does not affect *Streptococcus* and *Clostridium* populations (Williams *et al*, 2013). A phylogenetic tree showed *Clostridium* from plants and gut intermingled indicating that gut bacteria were recently acquired or constantly exchanged (Fig. 2A). Recent work by Jin *et al* (2020) showed that bacteria and fungi colonizing different bamboo species appeared as gut colonizers after consumed by giant pandas. Interestingly, the greater the microbial diversity of bamboo, the greater the diversity found in faecal samples.

The cellulolytic activity of *Clostridium* as well as the presence of bacterial genes that encode plant cell wall degrading enzymes (endocellulase, β‐glucosidase, xylan 1,4‐β‐xylosidase and endo‐1,4‐β‐xylanase) in panda guts highlight the microbial degradation of bamboo (Zhu *et al*, 2011; Guo *et al*, 2020) that could perhaps help to release endophytes during digestion.

Among the Proteobacteria that inhabit panda faeces, *Pseudomonas*, *Klebsiella, Enterobacter* and *Pantoea* are frequent endophytes of grasses and bamboo (Han *et al*, 2009b; Han *et al*, 2010; Liu *et al*, 2017). Most genes related to pathways for the plant secondary metabolite degradation have been associated with *Pseudomonas* from panda gut metagenomes (Zhu *et al*, 2018; Yao *et al*, 2019). *Klebsiella* and *Enterobacter* strains isolated from panda faeces carry genes involved in microbe‐plant interactions and cellulose degradation indicating their endophytic origin (Lu *et al*, 2015a, 2015b).

Weissellas from panda faeces are intermingled with plant weissellas in 16S rRNA gene phylogenetic trees, and this is the case with weissellas from human and bat faeces and from fish guts (Fig. 2B). Weissellas may contain tannases that would help to degrade tannins from plants (de Las Rivas *et al*, 2019).‐ Similarly, *Cryptococcus, Ramichloridium, Shiraia, Ceramothyrium, Rhinocladiella* and *Cephalosporium* are bamboo‐associated fungi detected in panda's faeces (Jin *et al*, 2020).

The gut microbiota of the giant panda resembles that of carnivorous and omnivorous bears because it has a different and lower diversity than other herbivores (Xue *et al*, 2015), but it also shows bacterial signatures that could result from being a bamboo specialist.

### Rabbits

Rabbits (*Oryctolagus cuniculus*) eat grasses, tree leaves and faeces. They are considered caecotrophagic animals because they ingest their soft faecal pellets produced by digestion in the caecum. Within herbivorous mammals, rabbits have the shortest mean retention time to digest their food, while the ruminants have the longest (Uden, *et al*, 1982; Kararli, 1995; Crowley *et al*, 2017). The rabbit digestive tract is adapted to process large amounts of fibre‐rich feed. Microbial fermentation of the food takes place in the caecum (Mackie, 2002; Harcourt‐Brown, 2004). Bacteroidetes dominate the caecal population and may be associated with the high fibre content in the diet (Crowley *et al*, 2017).

Like koalas, newborn rabbits in wild habitats ingest faecal pellets excreted by their mothers (Kovacs *et al*, 2006). When rabbits have access to the faecal excreta, bacteria colonize the caeca and rabbits have a reduced mortality after weaning in comparison to rabbits not consuming faeces (Combes et al., 2014).

*Cl* *ostridium, Anaerofustis, Blautia, Akkermansia* and *Bacteroides* are abundantly found in caecal samples, and in faeces *Oscillospira* and *Coprococcus* (Velasco‐Galilea *et al*, 2018). *Clostridium* as well as *Roseburia, Sutterella*, *Enterobacter* and *Desulfovibrio* have been reported as endophytes and in rabbit faeces (Velasco‐Galilea *et al*, 2018; Shanmuganandam *et al*, 2019). Interestingly, the genome of a gut *Enterobacter cloacae* strain has genes for plant colonization revealing characteristics of an endophyte (Shastry *et al*, 2020). *Roseburia* can produce butyrate, which is a nutrient for enterocytes (Tamanai‐Shacoori *et al*, 2017). Butyrate was identified as a promotor of gut barrier formation (Beaumont et al 2020).

### Herbivorous tortoise

Herbivory is not frequently found among reptiles (Yuan *et al*, 2015). However, there are herbivorous tortoises such as *Gopherus berlandieri* that is found in arid regions in Northeast Mexico and Southern United States (Judd and Rose, 1983). From the faeces of *G. berlandieri* healthy tortoises, we isolated *Klebsiella variicola* (Montes‐Grajales *et al*, 2019). *K. variicola* may be found as endophyte in maize, banana and rice plants (Rosenblueth *et al*, 2004) and was found as well in newborn baby faeces (Rosales‐Bravo *et al*, 2017). *K. variicola* has been proposed to be used as probiotic (Rosales‐Bravo *et al*, 2017) or crop inoculant. This bacterium is found in humans as an opportunistic pathogen (Martinez‐Romero *et al*, 2018).

From different *G. berlandieri* tortoises, distinct *K. variicola* isolates showed limited genetic diversity, suggesting that they are clones selected from a larger pool of these bacteria. Nitrogen‐fixing activity was detected by the acetylene‐reduction assay, both from faeces and *K. variicola* isolates. As tortoises are coprophagous, it seems possible that they acquire *K. variicola* tortoise‐borne strains directly from their mother or from the faeces of other tortoises. We surmise that *K. variicola* in tortoises were plant‐borne but we did not find them in their vegetal food; thus, they are not continuously ingested. Some *K. variicola* clones seem to have become stable microbiota, selectively maintained in tortoises by coprophagy. *Clostridium* was abundantly found in *Gopherus flavormarginatus* (Garcia‐De la Peña et al., 2019), *Gopherus polyphemus* (Yuan *et al*, 2015) and *G. berlandieri* faeces.

### Stinkbugs

Some stinkbugs from the superfamilies Coreoidea, Lygaeoidea and Pyrrhocoroidea feed on sap of diverse plants that have burkholderia endophytes. *Burkholderia* were found in insect guts in specialized compartments with crypts that have restricted entry and high burkholderial densities (Kikuchi *et al*, 2007, 2011; Kim and Lee, 2015). Recently, two novel genera were named for burkholderia subclades, *Paraburkholderia* and *Caballeronia* (Sawana *et al*, 2014; Dobritsa and Samadpour, 2016), and these are differentially encountered in stinkbug families that have specialized diets (Takeshita *et al*, 2015; Takeshita and Kikuchi, 2020). The symbiosis of phytophagous stinkbugs with these bacteria seems to be ancient (Kikuchi *et al*, 2011; Takeshita *et al*, 2015). Symbionts are beneficial to the insects, as stinkbugs without them displayed developmental delays, impaired survival or reduced size (Kikuchi *et al*, 2007; Boucias *et al*, 2012a; Xu *et al*, 2016a). Bacteria may confer stinkbugs resistance to the insecticide fenitrothion (Kikuchi *et al*, 2012; Tago *et al*, 2015). A transcriptome analysis of midgut‐colonizing *Burkholderia insecticola* from the bean‐bug *Riptortus pedestris* showed that bacteria recycle the host nitrogen wastes allantoin and urea, provide B vitamins, especially B12 and supply methionine and tryptophan to the host (Ohbayashi *et al*, 2019). Burkholderias constitute nice examples of plant bacteria that become gut colonizers, which are acquired by each new insect generation (Kikuchi *et al*, 2007; Kikuchi *et al*, 2011). Consequently, reported phylogenies showed that insect‐gut and plant burkholderias are intermingled (Itoh *et al*, 2014; Tago *et al*, 2015; Xu *et al*, 2016b **)**.

### Carmine cochineal

Carmine cochineals (*Dactylopius* spp.) are a group of hemipteran insects that have cultural and economic importance, as they produce a pigment called carminic acid that is used in industries like food, cosmetics and textiles. They originated in Mexico and South America (Chávez‐Moreno et al., 2009; Chávez‐Moreno et al., 2011; Mazzeo *et al*, 2019). Cochineals are sap‐suckers of *Opuntia* and other cactuses (Chávez‐Moreno *et al*, 2009). Our first microbial diversity study of different carmine cochineal species performed by Ramírez‐Puebla *et al* (2010) using PCR‐product sequencing, showed that the insect symbionts were related to plant endophytes such as *Herbaspirillum*, *Acinetobacter* and *Mesorhizobium*. Further metagenomic studies of *D. coccus* and *D. opuntiae* allowed us to obtain the genome of a betaproteobacterium (*Dactylopiibacterium carminicum*) related to the grass endophytes *Uliginosibacterium* and *Azoarcus* (Vera‐Ponce de León *et al*, 2017). Several characteristics of *Dactylopiibacterium* remind endophytes, like the ability to fix atmospheric nitrogen, to produce cellulases and pectinases, and to catabolize salicylic acid that is produced in plants (Vera‐Ponce de Leon et al 2017; Bustamante‐Brito *et al*, 2019). Our results suggested an endophytic origin of *Dactylopiibacterium* symbionts.

In the carmine cochineal guts, we found one species of endophytic fungi belonging to the genus *Coniochaeta*. *Coniochaeta* species are main endophytes of trees and grasses with some species well known for their production of a broad spectrum of antimycotics (Xie *et al*, 2015).

### Bark beetles and weevils

Wood‐eating *Dendroctonus* beetle guts contain nitrogen‐fixing bacteria, for example *Raoultella terrigena* that is a common endophyte (Morales‐Jiménez *et al*, 2013). Endophytes that have been used as plant growth‐promoting bacteria were also isolated from *Dendroctonus* beetles. Among these, *Serratia, Pseudomonas* and *Rhanella* species were able to recycle uric acid (Morales‐Jiménez *et al*, 2013). An additional study using 16S rRNA gene identification of gut bacteria from the pine‐pest *Monochamus alternatus* (Coleoptera) showed *Enterobacter, Raoultella, Serratia, Lactococcus* and *Pseudomonas,* which are commonly found in plant tissues as well. *Enterobacter* was the most common in larval and *Serratia* in pupal intestines. These bacteria may help to degrade the terpene pinene found in pines (Chen et al 2020).

Weevils are important beetle pests of stored grain legumes that feed and reproduce on dried seeds (Tuda, 2007). The neotropical genus *Acanthoscelides* comprises a diverse group of weevils some specialized on *Phaseolus* seeds (Alvarez *et al*, 2005). We studied the gut microbiota of bean weevils, *Acanthoscelides obtectus* (Coleoptera: *Chrysomelidae*, *Bruchinae*). Weevils were collected from inside wild *P. vulgaris* seeds from vines growing in mountain fields in the state of Morelos, Mexico. 16S ribosomal RNA genes from midgut DNA or from isolates were sequenced to generate a census of bacterial communities. We identified bacteria related to *Agrobacterium*, *Bacillus, Massilia, Gluconacetobacter, Propionibacterium, Asaia* and *Bradyrhizobium*. *Bacillus* isolates were frequently identified in bean seeds (Lopez‐Lopez *et al*, 2010) and leaves as endophytes (de Oliveira Costa *et al*, 2012). We suppose that *P. vulgaris* endophytes are transferred to the guts of *A. obtectus* weevils when they feed on bean seeds. Weevil insects in turn may transfer these bacteria to other plants and seeds.

### Maguey red worm

The maguey red worm (*Comadia redtenbacheri*) is edible and endemic to Mexico. Larvae are plant‐eating specialists of *Agave* inner plant tissues (Hernández‐Flores *et al*, 2015; Cárdenas‐Aquino *et al*, 2018). *Enterococcus* and *Klebsiella* that secrete indole‐acetic acid and solubilize phosphate were isolated as endophytes from leaf bases of agave plants (Martinez‐Rodriguez *et al*, 2019) and from the larva guts as well (our unpublished results). In the larvae from other Lepidoptera, *Spodoptera littoralis* an antimicrobial peptide was found secreted by *Enterococcus* located on the gut epithelium (Shao *et al*, 2017). Furthermore, microbiomes from distinct agave plants have been reported (Coleman‐Derr et al., 2016; Martinez‐Rodriguez et al., 2019; Flores‐Nuñez et al 2020) and several of the bacterial genera encountered therein were also identified in red worm guts (Hernández‐Flores *et al*, 2015) and from gut microbiomes in our laboratory.

*Cellulosimicrobium* found in cactus has an outstanding capability to degrade the plant cell wall using cellulases, xylanases and pectinases (Han *et al*, 2009a). *Cellulosimicrobium* was isolated from elephant and alpaca faeces (Jiang *et al*, 2013). We identified this bacterium from maguey worm microbiomes. Isolated strains from the larva guts of another lepidoptera, *Plutella xylostella* were capable of degrading plant phenolic compounds (Xia *et al*, 2017).

### Monarch butterflies

Monarch butterflies have a specialized plant diet. Milkweeds of the genus *Asclepias* are the preferred food of monarch caterpillars and adult monarchs feed on the nectar and pollen of flowers that provide sugars and other nutrients. We analysed the overwintering microbiota from guts of adult monarch butterflies, *Danaus plexippus* (Lepidoptera: *Nymphalidae*, *Danainae*) by sequencing metagenomes and 16S rRNA genes from bacterial isolates. We identified 16S rRNA gene sequences for *Asaia*, *Pseudomonas*, *Serratia*, *Enterococcus*, *Carnobacterium*, *Kinetoplastibacterium*, *Xylophilus*, *Polaromonas*, *Herbaspirillum* and *Lactococcus* bacteria. Some of these bacteria such as *Pseudomonas, Serratia, Enterococcus*, and *Herbaspirillum* are well known endophytes of plants. In guts of adult monarch butterflies, the acetic acid bacterium *Commensalibacter* was the most abundant (Servín‐Garcidueñas et al., 2014; Servin‐Garcidueñas and Martínez‐Romero, 2014).

### Cabbage white butterfly

The adult cabbage white butterflies (*Pieris rapae*) contain *Enterobacter* as well as *Flavobacterium* in their guts (Steinhaus, 1941); in the larvae, species from the genera *Asaia, Acinetobacter, Methylobacterium, Enterobacter* and *Pantoea* have been found (Robinson *et al*, 2010a, 2010b). Bacteria from these genera are common plant endophytes. Sinigrin, a glucosinolate found in Brussels sprouts, affects the bacterial community composition when fed to the cabbage white butterfly larvae. The bacteria found in the midgut may participate in sinigrin degradation (Robinson *et al*, 2010a, 2010b).

### Drosophila and fruit flies

*Lactobacillus plantarum* is found in plants as endophyte (Minervini et al., 2018) and commonly in *Drosophila* gastrointestinal tracts (Siezen *et al*, 2010). *L. plantarum* from *Drosophila* is related to strains from the same species from human faeces and plants (Fig. 2C). *Drosophila* in natural habitats may feed on plants but mainly on yeast from fermented fruits (Becher *et al*, 2012). *L. plantarum,* considered a facultative symbiont, is continuously ingested and excreted by *Drosophila* (Storelli *et al*, 2018). It provides acetyl‐glutamine to the host, produces a hormone‐signalling control (Storelli *et al*, 2011) and stimulates the production of intestinal peptidases (Matos *et al*, 2017). Notably, an improvement of host beneficial effects was obtained by an experimental evolution assay of *L. plantarum* across 20 *Drosophila* generations (Martino *et al*, 2018).

Similarly, from the Mediterranean fruit fly several enterobacteria were isolated including *Klebsiella* that was found in all samples; nitrogen fixation was detected in the enterobacterial cultures (Behar *et al*, 2005). An additional study from our laboratory (using 16S rRNA gene identification of cultured gut bacteria) from wild larvae from the Mexican fruit fly *Anastrepha ludens* showed *Bacillus, Lactobacillus, Pseudomonas, Enterobacter, Klebsiella, Acinetobacter, Leuconostoc* and *Weissella*. All of them are reported endophytes. Nitrogen‐fixing activity was detected by the acetylene‐reduction assay with *Klebsiella* and *Enterobacter*. Pectinolytic activity was observed in *Pseudomonas* and *Bacillus* and uricolytic activity in *Pseudomonas*. Additionally, we isolated in culture *Pichia* and *Hanseniaspora* yeasts (that are known fungal endophytes) from *A. ludens* larvae. *Pichia* was present in the oranges where larvae were feeding. Both yeasts were found in *Drosophila suzukii* (Hamby *et al*, 2012). *A. ludens* is an important pest in Mexico and Central America that attacks several fruit species especially citrus and mangos. Larvae feed on the fruits causing great losses, and biological control with sterile males has been used to control this pest (Barker et al., 2013).

## An endophytic‐enteric‐soil‐endophytic cycle

**Fig. 3:**
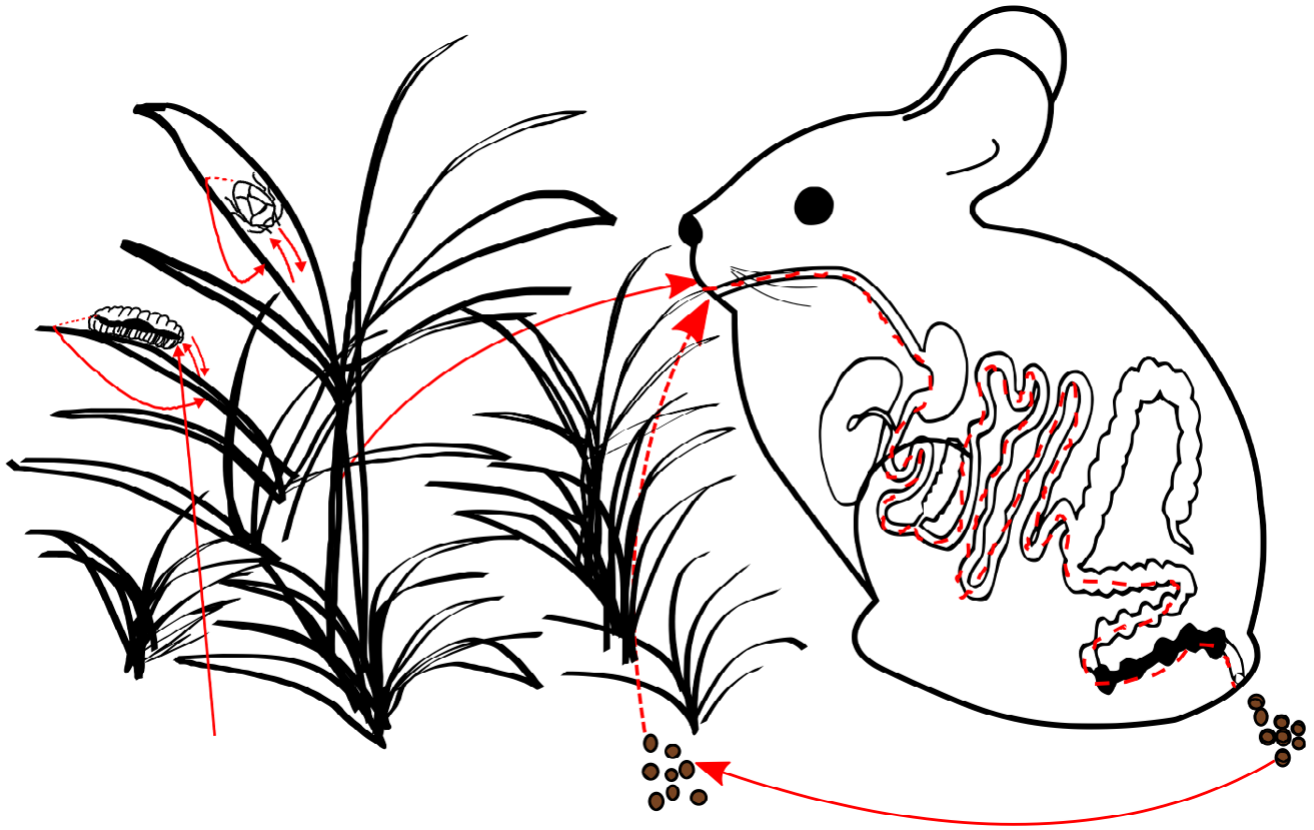
Schematic representation of the endophytic‐enteric microbiota cycle. Inside plant‐tissue endophytes may gain access to animal guts when animals eat plants, in turn animals produce faeces that may carry the ingested plant bacteria that would be available to colonize plants again. Coprophagy (faeces ingestion) is a shortcut in the cycle, as well as the transfer of insect bacteria to the plant during feeding or direct acquisition of bacteria from soil.

We showed data to support the endophytic origin of some gut bacteria, and we propose here the natural existence of an endophytic‐enteric‐soil‐endophytic microbiota cycle, designated hereafter as endophytic‐enteric cycle (Fig. 3) in which plant tissues could act as enteric ‘fibre capsules’ to protect plant endophytes from being digested in the stomach and allowing their later release in the intestine. If plant‐borne microbes from guts pass to the environment, then they may become soil bacteria and a natural biological inoculant of plants. By colonizing plants, these bacteria would complete a microbe cycle. Common ecological features in roots and gut (Ramírez‐Puebla *et al*, 2013) support their sharing bacteria. A shortcut in the endophytic‐enteric cycle would be faecal ingestion (coprophagy), which is a common practice among many animals, we surmise that animals were pioneers on faecal transplants. Fecal transplants are successfully used in modern Medicine by donating processed faeces from healthy humans to human patients with chronic gut diseases (Petrof and Khoruts, 2014).

Endophytes, which may or may not reproduce in the gut, could pass in faeces to soil and water. Tracing bacteria from animal guts to the soil was made possible by identifying antibiotic‐resistant bacteria in soil that derived from manure obtained from chicken or cow faeces (Wichmann *et al*, 2014). Spread of enteric bacteria could lead to a flow of antibiotic resistance genes from sewage, manure or slurry to humans (Linton, 1986). Farmland soils that were fertilized with chicken manure had high levels of antibiotic‐resistant bacteria (Zhao *et al*, 2017), and the impact of the use of antibiotics in farm animals has been studied (Qian *et al*, 2018; Heuer et al., 2011). Manure would constitute not only a way to recycle large amounts of N to plants but a source of bacteria for plants, and this has been shown when plants got contaminated with pathogens from animal manures (Guan and Holley, 2003). It was reported that human health depends on plant health, which in turn is determined by soils (Hirt, 2020).

As soils are rich in bacteria, insect microbiota may derive directly from soil (Fig. 3) and indeed soils were the main source and not host plants for the leaf‐eating caterpillar microbiota (Hannula *et al*, 2019). This may be considered as another shortcut in the cycle and remains to be tested with other insects. Certainly, there is a very dynamic flux of microbes to the gut. In the reverse direction, phytophagous insects that harbour multiple gut bacteria in oral secretions may transfer microbes to plants during feeding (Fig. 3) (Chung *et al*, 2013) and this is how many insects are vectors of plant pathogens.

Endophytes normally feed on plant products, and their degradative capabilities could be of use in catabolizing plant‐derived nutrients or suppress pathogens and fix nitrogen in the gut, as they do in plants. Gut bacteria use plant fibres (dietary fibre) to produce short‐chain fatty acids (such as butyric acid) (Baxter et al., 2019) that are intestine cell (enterocyte) nutrients (Ríos‐Covián et al., 2016; Wang *et al*, 2019).

Not all plant bacteria would survive digestion and participate in the endophytic‐enteric cycle. Therein, spore‐producing bacteria may be particularly successful, as spores are resistant forms which could even become activated inside guts. Notably, spores may be an important constituent of gut microbiomes (Browne *et al*, 2016).

## Critical issues

Even though there is clear evidence of plant‐derived bacteria in guts, a major question remains, are all small fragments of plant tissues effectively removed before macerating faecal samples? If not, they could be a source of DNA that would not reflect true gut bacteria. Work with panda faeces (Wei *et al*, 2007; Xue *et al*, 2015; Jin *et al*, 2020) addressed this issue, but it is not the case in all studies with faeces microbiota. Caution should be taken with metagenomic data, and microbial cultures from guts not to erroneously consider bacteria or fungi in plant fragments as *bona fide* gut microbiota. It is remarkable that most studies of gut microbiota are from faeces that may not well reflect gut bacteria (Zmora *et al*, 2018). Diet bacteria should be analysed concomitantly with gut microbiota and a comparison of the survival in the digestive tract of plant‐surface bacteria (Leff and Fierer, 2013) in relation to endophytes would be highly informative.

Seemingly, carnivores have endophytes in their guts as well. For example, *Prevotella*, which is known to be able to degrade plant derived carbohydrates, was the most abundant bacterium in the feline gut (Alessandri *et al*, 2020b). Endophytes in carnivore guts may derive from ingesting herbivore guts or faeces or soil‐contaminated meat. Notably, the gut microbiota of cats and dogs is determined as in humans by the diet and modified by overweight or inflammatory diseases (Alessandri *et al*, 2020a).

We suppose that plants may protect themselves from herbivory by possessing not only antimetabolites and toxins (in many cases produced by microbes) but also microbes that may cause disease or death to herbivorous animals when ingested. In contrast, plant bacteria may be useful, for example, *Lactobacillus plantarum* that is found in human and in omnivore gastrointestinal tracts (Siezen *et al*, 2010; Endo *et al*, 2010) is used as a probiotic (Panigrahi *et al*, 2017; Raveschot *et al*, 2020), which may help animals to better resist viral infections (Kumar *et al*, 2010; Kikuchi et al 2014), like other endophytic‐enteric bacteria do when used as probiotics (Chai *et al*, 2013; Mahooti et al 2020; Baud *et al*, 2020). However, probiotics constitute only transient members of the gut microbiota, remaining short periods in the gastrointestinal tract (Zmora *et al*, 2018) as we suppose some endophytes would do. Additionally, *L. plantarum* and *Weissella* are used in fermented food from plant products (Wacher‐Rodarte et al., 2015, Kavitake *et al*, 2016). Curiously, a metabolomic study showed that fermented food and stools were similar (Quinn *et al*, 2016).

Herbivores have most probably picked up their stable microbiota from plant‐associated bacteria. Particularly, the evolutionary history of insects is tightly dependent on plants as food (McKenna and Farell, 2006). When an herbivore has recently ingested an endophyte, gut strains would be identical or very similar to the plant isolates, as can be observed in phylogenies from *Weissella* from panda faeces, in *L. plantarum* and clostridia phylogenies (Fig. 2) or in burkholderias from the stinkbugs (Itoh *et al*, 2014; Tago *et al*, 2015; Xu *et al*, 2016b). On the other hand, when the endophytes coevolved with their animal hosts which maintain them by vertical transfer, then these bacteria would be divergent from plant bacteria, as observed in some insect symbionts such as *Commensalibacter* and *Dactylopiibacterium* (Servín‐Garcidueñas et al., 2014; Vera‐Ponce de León *et al*, 2017). Specialized insect endosymbionts in abdominal bacteriomes could have derived from gut bacteria, which in turn derived from endophytes that were found in plants that may not exist today. Flavobacterial endosymbionts of scale insects (Rosenblueth *et al*, 2012, 2018), which are around 200 million years old and provide essential amino acids to the insect, perhaps derived from plant flavobacteria which are found mainly in wild but not in domesticated plants (Pérez‐Jaramillo et al., 2018).

If we eat endophytes, we may excrete endophytes; this occurs with mealybug insects that eat and excrete honeydew containing *Gluconacetobacter* spp. (Ashbolt and Inkerman, 1990), a common nitrogen‐fixing endophyte from sugarcane (Caballero‐Mellado and Martinez‐Romero, 1994). Notably, the numbers of *Gluconacetobacter* bacteria diminish drastically in plants with nitrogen chemical fertilization (Fuentes‐Ramírez et al., 1999). Similarly, other agrochemicals may have strong effects on plant microbiota and thus on the endophytes which we ingest. Remarkably, fungal endophytes may vary depending on the habitat (Harrison and Griffin, 2020). Finally, inoculants used on plants should be considered not only in terms of their plant growth‐promoting capabilities but for their effects on human health as well. The use of cyanobacteria in plants should be carefully evaluated and avoided if possible. Cyanobacteria produce very harmful neurotoxins and hepatotoxins (Codd *et al*, 2005).

## Conclusions

Certainly all herbivores eat endophytes, some of them may be digested or not liberated from plant tissues but others may be active in the gut and contribute to fibre or tannin digestion or to the synthesis of essential amino acids or vitamins, fix nitrogen or provide defence against pathogens. When studying herbivore gut microbiota, plant fragments in guts should be independently analysed.

While stable members of the gut microbiota may have derived from the vegetal diet, newly ingested endophytes may be quite variable. It would be desirable to eat the ones that contribute most to human health either as probiotics or with food. There is still lots to explore on this considering the variable responses in human individuals to probiotics, and the large effects of crop management on plant microbes. Ideally, plant microbial inoculants should benefit both plants and humans. Clearly, the endophyte‐enteric cycle has relevance to animal health. So, which endophytes would you like to eat?

## Funding Information

This work was supported by grants from the National University of Mexico to EMR IN210021 and to LES‐G (PAPIIT‐DGAPA IA208019) to study the monarch microbiota.

## Conflict of interest

None declared.
